# How Do Tracking and Changes in Dietary Pattern during Adolescence Relate to the Amount of Body Fat in Early Adulthood?

**DOI:** 10.1371/journal.pone.0149299

**Published:** 2016-02-23

**Authors:** Bruna Celestino Schneider, Samuel de Carvalho Dumith, Carla Lopes, Milton Severo, Maria Cecília Formoso Assunção

**Affiliations:** 1 Post-Graduate Program in Epidemiology, Federal University of Pelotas, Pelotas, Brazil; 2 Post-Graduate Program in Public Health, Federal University of Rio Grande, Rio Grande, Brazil; 3 Public Health Institute, Department of Clinical Epidemiology, Preventive Medicine and Public Health, University of Porto Medical School, Porto, Portugal; University of Leipzig, GERMANY

## Abstract

**Background:**

Few studies have addressed the influence of dietary patterns (DP) during adolescence on the amount of body fat in early adulthood.

**Objective:**

To analyze the associations between DP tracking and changes in the period between 15 and 18 years of age and the percentage of body fat (%BF) at age 18 years.

**Methods:**

We used data from 3,823 members of the 1993 Pelotas (Brazil) birth cohort. Body density was measured at age 18 years by air displacement plethysmograph (BOD POD) and the %BF was calculated applying the Siri equation. Based on the estimates from the FFQ, we identified DP at ages 15 (“Varied”, “Traditional”, “Dieting” and “Processed meats”) and 18 years (“Varied”, “Traditional”, “Dieting” and “Fish, fast food and alcohol”). The DP tracking was defined as the individual’s adherence to the same DP at both ages. Associations were tested using multiple linear regression models stratified by sex.

**Results:**

The mean %BF was 25.0% (95% CI: 24.7 to 25.4), significantly greater for girls than boys (p<0.001). The adherence to any DP at age 15 years was not associated with the %BF at age 18 years. However, individuals who adhered to a “Dieting” DP at age 18 years showed greater %BF (1.30 and 1.91 percentage points in boys and girls, respectively) in comparison with those who adhered to a “Varied” DP. Boys who presented tracking of a “Dieting” DP presented greater average %BF in comparison with others DP, as well as girls who changed from the “Traditional” or “Processed meats” DP to a “Dieting” DP.

**Conclusion:**

These results may support public health policies and strategies focused on improving dietary habits of adolescents and young adults and preventing accumulation of body fat, especially among the adolescents with restrictive dietary habits.

## Introduction

Obesity is defined as the abnormal or excessive fat accumulation, ultimately caused by a positive energy balance [1]. This event constitutes a major risk factor for several chronic diseases, such as diabetes and cardiovascular diseases [2, 3]. In 2012, over 40 million children aged more than 5 years around the world were overweight or obese [4]. In Brazil, data from the family budget survey (POF) [5] carried out in over 50,000 households across the country showed that the prevalence of overweight or obesity in adolescents reached 21.5% in 2009, and obesity was higher in boys (5.9%) than in girls (4.0%).

Adolescence is a period between the ages of 10 and 19 years, characterized by important physiological and behavioral changes [6]. During this period, the amount of body fat (BF) tends to increase, especially in girls, driven by the sexual steroids hormones. The exposure to an adverse lifestyle in adolescence, such as inadequate eating behaviors, may lead to an increased accumulation of BF, independently of genetic and environmental factors [2, 3, 7].

Several methods have been applied in order to investigate the association between diet and BF, mainly aiming to identify dietary profiles and patterns rather than measuring nutrient intake adequacy. Particularly, the dietary pattern (DP) analysis allows a broad evaluation of food consumption, considering possible antagonistic and synergistic relationships among the nutrients [8–10], because people usually do not eat isolated foods or nutrients but combinations of several foods, nutrients and other components. The dietary pattern approach is useful in examining the relationship between diet and several health outcomes because to examine food intake globally [10]. The tracking or changes in the DP throughout life, especially during adolescence and early adulthood, may have a direct or indirect impact on body composition [11]. Moreover, previous studies have shown that inadequate dietary habits acquired during adolescence are likely to persist throughout adulthood [12].

Few studies have investigated the relationship of the tracking and changes in DP with the amount of BF during adolescence and early adulthood, based on prospectively collected data. We are not aware of any study carried out in Brazil in the published literature on this topic. In this context, we aimed to investigate the associations of DP tracking and changes in the period between 15 and 18 years of age with the %BF at age 18 years in a middle-income setting.

## Methods

### Study population

This is a prospective longitudinal study carried out with data from the 1993 Pelotas (Brazil) birth cohort, which is a population-based study including all hospital births from mothers living in the urban area of Pelotas from January 1^st^ to December 31^st^ 1993 (n = 5,320). Accounting for deaths and refusals, the original cohort comprises 5,249 children (98.7% of all births). More details about the cohort can be found elsewhere [13, 14]. In this study, we used data collected at ages 15 and 18 years, for which all the members of the original cohort were eligible.

### Dependent variable

Body density was estimated at age 18 years by air displacement plethysmograph (BOD POD), applying a standardized technique [15, 16]. The percentage of body fat (%BF) was determined using the Siri equation [17] (%G = [(4.95/D)– 4.50] x 100), where “D” is the body density (D = mass/volume). Participants wore minimal fitting clothing (spandex shorts and tops) and a swim cap. In order to ensure quality control, the equipment was calibrated on a daily basis according to the supplier recommendations.

### Independents variables

#### Dietary patterns (DP)

Food Frequency Questionnaires (FFQ) were administered at ages 15 and 18 years, with recall period of 12 months. These FFQ were adapted from a previously validated questionnaire to the Brazilian population [18]. At age 15 years, trained interviewers applied a qualitative FFQ including 81 food-items and the frequency of consumption of each food (one to ten times per day/week/month/year) was estimated. At age 18 years, we used a semi-quantitative self-reported FFQ, available in electronic format and comprised by 88 food-items, assessing the categorized frequency of consumption (never or less than 1 time/month, 1 to 3 times/month, 1 time/week, 2 to 4 times/week, 5 to 6 times/week, 1 time/day, 2 to 4 times/day, 5 times/day or more). In this study, we only included those food-items assessed in both FFQs (n = 79).

**Fig 1:**
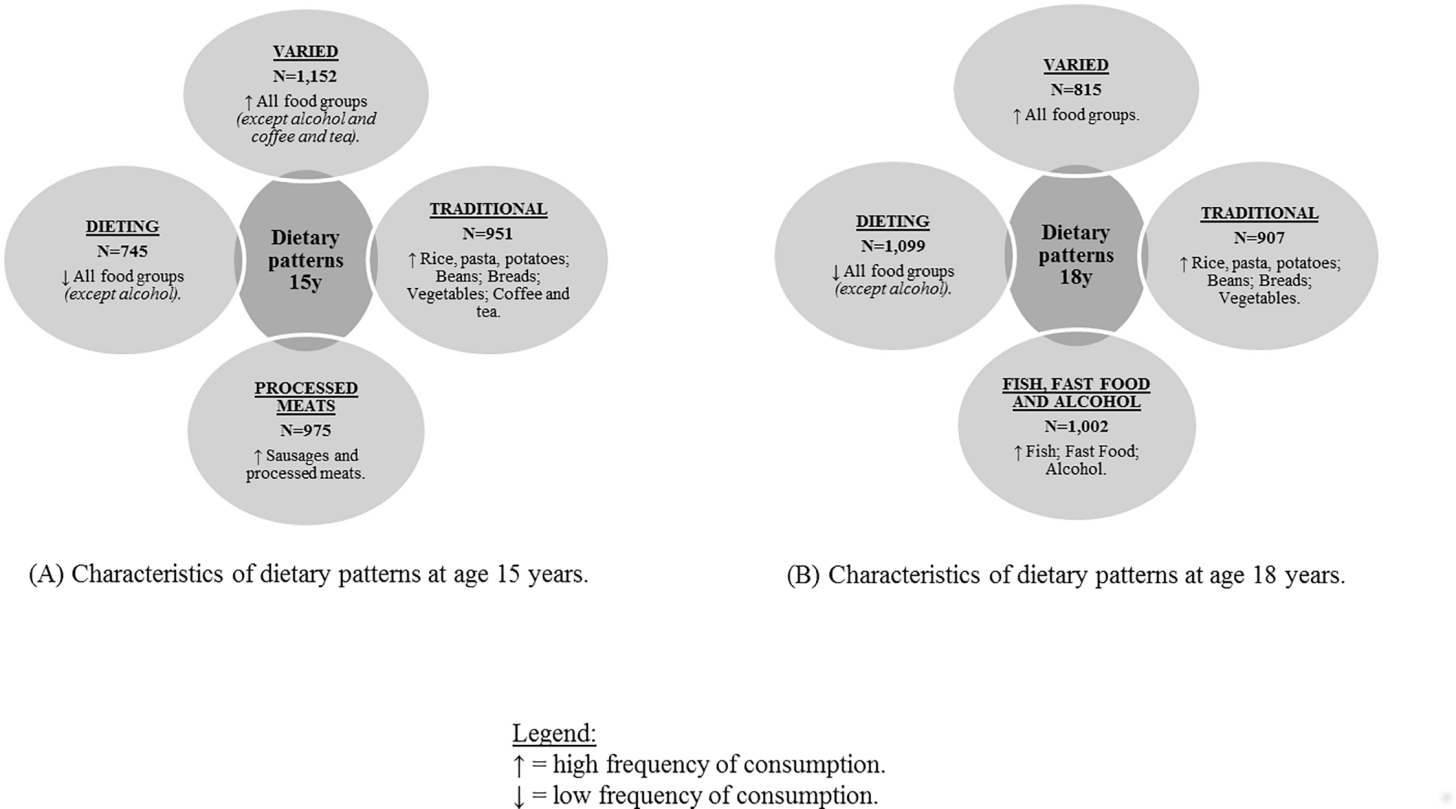
Dietary patterns. (A) Characteristics of dietary patterns at age 15 years. (B) Characteristics of dietary patterns at age 18 years.

The 79 food-items available in both FFQs were divided into 19 foods groups according to their nutritional characteristics (Table 1) were used to construct the DP at both ages. Four DP were identified at ages 15 (Fig 1A) and 18 years (Fig 1B): “Varied”, “Traditional”, “Dieting” at both ages; the “Processed meats” DP only at age 15 years; and the “Fish, fast food and alcohol” DP at 18 years of age only. The “Varied” DP presented a high frequency of consumption of most of the 19 food groups; conversely, the “Dieting” DP is characterized by the low consumption of most food groups. The “Traditional” DP was marked by the frequent consumption of food groups that constitute the Brazilian traditional diet. The “Processed meats” and the “Fish, fast food and alcohol” showed a high frequency of consumption of its respective food groups, in comparison with the whole sample.

**Table 1:** Food groups of the dietary patterns.

Label of group	Foods
*Rice*, *pasta*, *potatoes*	Rice + pasta + potatoes + manioc cassava
*Beans*	Beans + lentil
*Breads*	Brown bread + white bread + bread shape
*Vegetables*	Letucce + tomato + carrot + pumpkin + cole + cabbage + chayote + natural cucumber + string bean + beet + cauliflower + garlic + onion
*Fruits*	Banana + orange or mandarine + papaya + pineapple + alligator pear + mango + peach + guava + pear + apple + watermelon or melon + strawberry + grape + natural fruit juice
*Snacks*	Chips + cracker + cake + popcorn + pies + sweet biscuits
*Sweets/Candies*	Ice cream + candies + desserts + chocolate
*Soft drinks and sugary beverages*	Soft drink + light soda + artificial juice
*Fast Food*	Cheeseburguer + hot dog + pizza + fried polenta + fried cassava + french fries + mayonnaise + canned foods + canned fish
*Sausages and processed meats*	Ham, mortadella or salami + dried meat + sausage
*Fish*	Fish + shrimp
*Chicken*	Chicken
*Viscera*	Viscera
*Red meat*	Beef + carcass meat + pork
*Eggs*	Eggs
*Milk and dairy products*	Milk + yogurt + cheese
*Added sugar and chocolate powder*	Sugar + chocolate powder
*Alcohol*	Wine + whiskey + beer
*Coffee and tea*	Coffee + tea

### Potential confounders

#### Sociodemographic characteristics

All sociodemographic variables were collected at age 15 years. Sex (boy/girl) and skin color (white/non-white) were observed and registered by a trained interviewer. Maternal and paternal education levels (years of complete education) were reported by the adolescent. We also calculated the wealth index, which is a score that takes into account the ownership of selected assets collected through a questionnaire, using principal component analysis [19]. The first component was extracted and categorized into quintiles. The first (Q1) and the fifth (Q5) quintiles represent the poorest 20% and the richest 20%, respectively.

#### Body mass index

Weight and height were measured by trained anthropometrists using standard methods. At age 15 years, weight was measured to the nearest 0.1 kg using a calibrated digital scale (UM-080 scale, TANITA, Tokyo, Japan). At age 18 years, weight was measured to the nearest 0.1 kg using an electronic scale attached to the BOD POD system. At both ages, height was measured to the nearest 0.1 cm using a portable stadiometer. The body mass index (BMI) for age and sex was calculated according to the WHO recommendations [20] and classified using the following cut-off points: overweight (BMI-for-age >+1SD and <+2SD) and obesity (BMI-for-age > +2SD).

#### Physical activity

The International Physical Activity Questionnaire (IPAQ) [21], validated for the Brazilian population [22], was applied to assess habitual physical activity during the seven days prior to interview at age 15 years. We used the short interview-administered version composed by seven items, which covers three domains of physical activity: transportation, household/gardening and leisure-time activities. The number of days/week and the time spent on physical activities per day from all three domains were recorded. Those individuals who referred spending 300 minutes or more in physical activities per week were considered as active; complementarily, in this study, those who referred spending less than 300 minutes/week in physical activities were considered as insufficiently active [23].

### Statistical analyses

Initially, we estimated the daily frequencies of consumption of each food group and observed skewed distributions for most of them, as well as high percentage of non-intake. Therefore, we opted to categorize the frequency of consumption into tertiles.

The DP were generated using latent class analysis (LCA), which is used to identify distinct groups of individuals from a sample that cannot be observed directly (latent classes), which can be originated from the observed variables [24]. It is based on statistical models that are able to compute the probabilities of belonging to a specific class for each subject. In this study, the latent classes were labelled as DP. The number of DP was defined according to the Bayesian Information Criterion (BIC). The model with the lowest BIC was selected in order to optimize the fitness of the model. The best solution was identified when the increase in the number of classes did not lead to a decrease in the BIC. Based on this modelling, the preference was for a four-class solution. All LCA models were fitted using the software MPlus version 5.2.

In order to label the DP, we identified the food groups more frequent in each DP and compared the proportion of adolescents in the lowest and highest categories of frequency of consumption. When the difference in the highest category was equal to or greater than approximately +10 percentage points, we assumed that the DP was characterized by the high consumption of that specific food group. Similarly, differences equal to or greater than approximately +10 percentage points in the lowest category of frequency of consumption were interpreted as low consumption of the specific food group in the DP.

All cohort members with valid information on diet at ages 15 and 18 years and %BF at age 18 years (N = 3,823) were included in the present analysis. Data management and analysis were carried out using Stata (version 12.1). All analyses including the %BF were adjusted for height [25]. %BF means and respective 95% confidence intervals were presented. In crude analysis, statistical comparisons were made by applying analysis of variance (ANOVA) and nonparametric tests for trend (nptrend) were performed. We used multiple linear regression models to obtain adjusted β coefficients and standard errors (SE), and modelling process followed standard procedures. In order to obtain independent associations between DP (tracking and changes) and %BF, analyses were controlled for the following potential confounders: sex, skin color, education level of the head of the household, wealth index, physical activity age 15 years and BMI at age 15 years. All analyses were stratified by sex and a 5% significance level was adopted.

### Ethical aspects

This study is part of the project entitled "Early and contemporary influences on body composition, human capital, mental health and chronic disease precursors in complex cohort born in 1993 in Pelotas, Brazil", which was approved by the Ethics Committee in Research of the School of Medicine of the Federal University of Pelotas (process number 05/11) and all participants signed an informed consent agreeing to participate in the study. When the participant was under 18 years, the signed consent was obtained from the parent/responsible.

## Results

### Characteristics of the participants

Of the 3,823 adolescents analyzed in the present study, the majority was female (51.4%), with white skin color (64%), from households with head formally educated for five to eight years (42.6%) and were insufficiently active (53.4%). About 30% of the participants had excess body weight (overweight or obese) (data not shown in Table).

### Dietary Patterns, Tracking and Changes

At age 15 years, the “Varied” DP was the most frequent (30.1%; 95%CI: 28.7 to 31.6%), followed by the “Processed Meats” DP (25.5%; 95%CI: 24.1 to 26.9%), the “Traditional” DP (24.9%; 95%CI: 23.5 to 26.2%) and the “Dieting” DP (19.5%; 95%CI: 18.2 to 20.7%).

At age 18 years, the “Dieting” was the most adhered DP (28.7%; 95%CI: 27.3 to 30.2%), followed by the “Fish, fast food and alcohol” (26.2%; 95%CI: 24.8 to 27.6%), the “Traditional” (23.7%; 95%CI: 22.4 to 25.1%), and the “Varied” (21.3%; 95%CI: 20.0 to 22.6%) DP.

Tracking was more frequent for the “Dieting” DP (36.2%), especially among girls and the wealthiest adolescents. The most frequent change was from the “Processed Meats” DP at age 15 years to the “Dieting” DP (38.1%) at age 18 years.

### Tracking and Changes of the Dietary Patterns and %BF

Table 2 presents the key features of the cohort members included in this study according to the %BF. The overall mean %BF was 25.0% (95%CI: 24.7 to 25.4%), greater in girls (32.7%; 95%CI: 32.2 to 33.2%) than in boys (16.0%; 95%CI: 15.5 to 16.6%). In boys, higher %BF was found among those with white skin color, from households with better formally educated head, and the richest 20%. Among girls, we found significant associations only with skin color and BMI. Being physically active at age 15 years was associated with lower %BF in boys (15.8%; 95%CI: 15.1 to 16.4) but not in girls.

**Table 2:** Means and respective 95%CIs of percentage body fat (%BF) by sociodemographic and behavioral characteristics.

Baseline variables (15y)	% Body Fat at age 18 years								
	Overall			Boys			Girls		
	N	Mean (95%CI)	p value	N	Mean (95%CI)	p value	N	Mean (95%CI)	p value
	3823	25.0 (24.7–25.4)		1858	16.0 (15.5–16.6)		1965	32.7 (32.2–33.2)	
Skin color			<0.0001*			<0.0001*			0.001*
* White*	2446	25.6 (25.2–26.0)		1195	16.5 (15.8–17.3)		1251	33.1 (32.5–33.7)	
* Non White*	1375	23.9 (23.4–24.5)		662	15.2 (14.3–16.1)		713	31.8 (31.0–32.7)	
Parental schooling			<0.0001^#^			<0.0001^#^			0.3^#^
* 0 to 4*	976	23.2 (22.5–23.9)		495	14.2 (13.3–15.2)		481	32.7 (31.6–33.8)	
* 5 to 8*	1621	25.2 (24.7–25.7)		778	16.6 (15.7–17.5)		843	33.0 (32.3–33.7)	
* 9 to 11*	837	26.0 (25.4–26.7)		408	17.0 (15.7–18.4)		429	32.5 (31.5–33.5)	
* 12 or more*	373	26.3 (25.4–27.3)		169	17.7 (15.8–19.5)		204	31.8 (30.6–33.0)	
Wealth index (quintiles)			<0.0001^#^			<0.0001^#^			0.5^#^
* 1 (lower)*	749	22.8 (22.0–23.6)		361	14.2 (13.1–15.2)		388	33.1 (31.8–34.4)	
* 2*	746	24.1 (23.3–24.8)		347	15.4 (14.2–16.6)		399	31.7 (30.5–32.8)	
* 3*	768	25.4 (24.7–26.2)		377	16.8 (15.6–18.1)		391	33.4 (32.3–34.5)	
* 4*	790	25.8 (25.1–26.5)		390	16.5 (15.2–17.8)		400	33.1 (32.1–34.1)	
* 5 (highest)*	756	26.6 (26.0–27.3)		375	18.1 (16.8–19.5)		381	32.3 (31.4–33.2)	
BMI (kg/m^2^)			<0.0001*			<0.0001*			<0.0001*
* Eutrofic*	2683	22.1 (21.7–22.4)		1269	13.0 (12.5–13.4)		1414	30.1 (29.6–30.5)	
* Overweight*	697	30.2 (29.5–31.0)		349	21.2 (20.0–22.3)		348	39.0 (38.0–40.0)	
* Obese*	324	37.7 (36.7–38.7)		186	30.8 (28.9–32.7)		138	44.4 (43.2–45.7)	
Physical Activity (minutes/week)			<0.0001*			<0.0001*			0.7*
* <300*	2040	26.7 (26.3–27.2)		697	16.5 (15.6–17.5)		1343	32.6 (32.0–33.2)	
* 300 or more*	1782	23.1 (22.6–23.6)		1160	15.8 (15.1–16.4)		622	32.8 (31.9–33.6)	

* Pearson's chi square test.

^#^ Linear tendency.

Crude and adjusted analyses of the association between DP and %BF at age 18 years are presented in Table 3. After adjustments, we did not observe any significant associations between the adherence to any DP at age 15 years and %BF. Conversely, adolescents who adhered to a “Dieting” DP at age 18 years showed, on average, a higher %BF than those who adhered to a “Varied” DP. At the same age, the adherence to a “Varied” DP was associated with lower %BF in comparison with those who adhered to the other DP.

**Table 3:** Crude and adjusted associations between dietary patterns at ages 15 and 18 years and percentage (%) body fat at age 18 years.

Dietary patterns	% Body Fat at age 18 years							
	Boys				Girls			
		Crude		Ajusted		Crude		Ajusted
	N	Mean (95%CI)	β (SE)*	β (SE)*	N	Mean (95%CI)	β (SE)*	β (SE)*
At age 15 years								
* Varied*	564	15.7 (14.7–16.7)	REF	REF	588	32.1 (31.2–33.0)	REF	REF
* Traditional*	498	15.2 (14.3–16.2)	-0.79 (0.54)	-0.37 (0.41)	453	32.5 (31.4–33.5)	0.28 (0.49)	0.17 (0.37)
* Dieting*	348	17.3 (15.9–18.7)	**1.54 (0.60)**	0.03 (0.48)	397	32.6 (31.7–33.5)	0.59 (0.51)	0.37 (0.40)
* Processed Meats*	449	16.7 (15.6–17.8)	1.00 (0.56)	0.17 (0.42)	526	33.5 (32.5–34.6)	**1.56 (0.47)**	0.57 (0.35)
At age 18 years								
* Varied*	422	14.4 (13.3–15.4)	REF	REF	393	30.8 (29.7–31.9)	REF	REF
* Traditional*	464	15.3 (14.3–16.3)	0.67 (0.59)	0.05 (0.44)	443	32.8 (31.7–33.9)	**1.18 (0.53)**	**0.87 (0.41)**
* Dieting*	489	18.4 (17.2–19.6)	**4.42 (0.58)**	**1.48 (0.45)**	610	34.2 (33.4–35.1)	**3.36 (0.50)**	**1.98 (0.39)**
* Fish*, *fast food and alcohol*	484	16.2 (15.1–17.2)	**2.05 (0.58)**	0.82 (0.45)	518	32.1 (31.3–33.0)	0.91 (0.52)	**1.32 (0.40)**

* *Model adjusted for skin color, parental schooling, wealth index, physical activity and body mass index at 15 years.* Bold values significant (p<0.05).

Associations of tracking and changes in DP between 15 and 18 years of age with %BF at age 18 years are shown in Table 4. After controlling for potential confounders, boys and girls who changed from a “Traditional” DP to a “Dieting” DP showed greater %BF than those who adhered to a “Traditional” DP at both ages (tracking). Boys who changed from a “Dieting” to a “Varied” or “Fish, fast food and alcohol” DP had significantly lower %BF at age 18 years. Among girls, changing from a “Traditional” DP to a “Fish, fast food and alcohol” DP, or from a “Processed meats” to a “Dieting” DP, was associated with greater %BF at age 18 years compared to those presenting DP tracking.

**Table 4:** Crude and adjusted associations between dietary patterns tracking and changes from 15 to 18 years of age and percentage body fat (%BF) at age 18 years.

Tracking and changes	% Body Fat at age 18 years							
	Boys				Girls			
	Crude			Ajusted	Crude			Ajusted
	N	Mean (95%CI)	β (SE)*	β (SE)*	N	Mean (95%CI)	β (SE)*	β (SE)*
Varied								
* Tracking*	186	15.2 (13.9–16.4)	REF	REF	180	31.3 (30.2–32.5)	REF	REF
* Change Traditional*	139	15.1 (13.7–16.5)	-0.20 (0.98)	-0.13 (0.76)	130	32.3 (30.9–33.7)	0.97 (0.90)	0.74 (0.70)
* Change Dieting*	89	18.6 (16.6–20.6)	**3.48 (1.12)**	1.19 (0.89)	108	33.8 (32.3–35.2)	**2.45 (0.96)**	0.90 (0.76)
* Change Fish*, *fast food and alcohol*	150	18.1 (16.7–19.5)	**2.80 (0.96)**	**1.62 (0.75)**	170	32.1 (30.9–33.2)	0.74 (0.84)	0.80 (0.65)
Traditional								
* Tracking*	149	14.8 (13.2–16.3)	REF	REF	152	31.2 (29.9–32.5)	REF	REF
* Change Varied*	118	13.5 (12.2–14.9)	-1.20 (1.09)	0.44 (0.78)	92	30.9 (29.5–32.3)	-0.33 (1.00)	-0.32 (0.77)
* Change Dieting*	127	18.4 (16.7–20.1)	**3.54 (1.07)**	**1.77 (0.78)**	134	34.7 (33.3–36.0)	**3.47 (0.89)**	**2.18 (0.69)**
* Change Fish*, *fast food and alcohol*	104	15.7 (14.1–17.3)	0.88 (1.13)	0.81 (0.81)	75	33.1 (31.7–34.6)	1.91 (1.06)	**1.93 (0.82)**
Dieting								
* Tracking*	117	20.4 (18.8–22.0)	REF	REF	153	33.7 (32.5–35.0)	REF	REF
* Change Varied*	46	16.2 (13.7–18.8)	**-4.16 (1.54)**	**-3.33 (1.20)**	41	30.6 (28.5–32.6)	**-3.20 (1.26)**	-0.16 (0.97)
* Change Traditional*	64	17.2 (15.1–19.4)	**-2.87 (1.39)**	-0.89 (1.07)	48	32.5 (30.4–34.7)	-1.24 (1.18)	-1.92 (1.05)
* Change Fish*, *fast food and alcohol*	121	17.1 (15.5–18.8)	**-3.29 (1.15)**	**-2.00 (0.87)**	155	32.5 (31.5–33.5)	-1.25 (0.81)	0.46 (0.65)
Processed Meats								
* Change Fish*, *fast food and alcohol*	109	16.9 (15.6–18.3)	REF	REF	118	31.2 (29.9–32.6)	REF	REF
* Change Varied*	72	15.4 (13.5–17.3)	-1.35 (1.28)	-0.09 (1.06)	80	31.9 (30.1–33.7)	0.61 (1.16)	-1.08 (0.87)
* Change Traditional*	112	16.2 (14.6–17.7)	-0.46 (1.14)	-0.66 (0.94)	113	34.2 (32.7–35.8)	**2.99 (0.92)**	0.83 (0.78)
* Change Dieting*	156	19.9 (18.4–21.4)	**3.19 (1.05)**	0.67 (0.88)	215	35.6 (34.5–36.7)	**4.37 (0.92)**	**2.11 (0.67)**

*Adjusted for skin color, parental schooling, wealth index, physical activity and body mass index at 15 years. Bold values significant (p<0.05).

## Discussion

This study assessed the relationship between tracking and changes of the DP in adolescence and body fat in early adulthood, using data from a large longitudinal study carried out in Southern Brazil since 1993. We found greater %BF was associated with DP tracking and changes from ages 15 to 18 years, mainly related to the maintenance of adherence to a “Dieting” DP or changing to a “Dieting” or a “Fish, fast food and alcohol” DP.

The significant associations between greater %BF and adherence to a “Dieting” DP at age 18 years or at both ages (15 and 18 years) could be explained by the fact that this DP was constructed based on the frequency of consumption rather than the amount of food consumed. Thus, adolescents who adhered to a “Dieting” DP could present infrequent consumption the majority of the food groups, but in large amounts. Consistent with our findings, a cross-sectional study carried out in Norway reported associations between the adherence and tracking to a “Dieting” DP, characterized by high intake of foods and drinks consumed during calorie-restriction (dieting), and increased risk of excess weight in adolescents aged 12–13 years [26]. Other possible explanation for the associations involving the tracking and changes in the “Dieting” DP may be related to the fact that diet does not have an isolated effect on body fat accumulation. This process is affected by multiple factors and results, ultimately, from a positive energy balance. There is evidence that regular physical activity helps controlling and decreasing the %BF during adolescence [27]. In preliminary analyses, we observed that adolescents who adhered to a “Dieting” DP at age 15 years were more likely to be physically inactive in comparison to those who adhered to the other DP. Although we have adjusted our analyses for the physical activity level at age 15 years, the possible changes in the patterns of this behavior were not considered, which could affect the associations between the DP and the %BF at 18 years.

We found that boys who changed from a “Dieting” to a “Varied” DP had significantly lower %BF at age 18 years. The “Varied” DP is characterized by the high frequency of consumption of all food items and, consequently, with higher variety of foods consumed. Although it is not possible to draw conclusions about the food intake, the “Varied” DP seems to be more balanced regarding energy and portion consumed. In Norway, Cutler (2011) [28] also reported inverse associations between adherence to a “Varied Norwegian” DP and risk of overweight in adolescents aged 12–13 years. In our study, individuals who adhered to a more restrictive DP in terms of frequency of food consumption at 15 years were probably those more concerned about health risks related to weight gain and, because of that, may have changed to a healthier lifestyle at 18 years, adhering to the “Varied” DP. Most subjects who adhered to a "Varied" DP at 18 years were physically active. Thus, these young adults may have increased their frequency of food consumption and energy expenditure without leading to an increase in the amount of body fat.

Among girls, we observed that changing from a “Traditional” to a “Fish, fast food and alcohol” DP was associated with greater %BF at age 18 years compared to those presenting DP tracking. Although eating fish has been recommended for preventing diseases, the consumption of fast foods has the opposite relationship. They constitute rich sources of saturated fatty acids and trans fatty acids and the frequent consumption could lead to adverse outcomes, such as obesity, hyperinsulinemia and insulin resistance [29, 30]. Studies have reported an increased consumption of fast foods during adolescence, reinforcing the need of promoting the adoption of healthy food choices [30]. The consumption of fast foods and alcoholic beverage characterize a highly energetic and minimally nutritious diet. Thus, those adolescents who have migrated from a "Traditional" DP to a “Fish, fast food and alcohol" DP may have increased their energy consumption, that could lead to a positive energy balance and, ultimately, to body fat accumulation. In boys, we also observed an increase in %BF associated with changing from a “Traditional” to a “Fish, fast food and alcohol” DP. Other studies have reported similar associations between fast food [29] and body composition among adolescents, but none of them used tracking or changing in food habit.

We acknowledge some limitations of our study. A different FFQ was applied to assess food consumption in each phase of the study; however, the same information were extracted from each of them in order to generate the DP, so that the instruments could be considered comparable. As the information on diet at age 18 years were collected using a self-applied FFQ, the precision and quality of the data may have been affected, leading to under- or overestimation of the frequency of food consumption. Individuals are more likely to misreport on their dietary habits at the beginning of adolescence than adults, as the awareness regarding health and diet tends to increase with age [9]. The method applied for generating the DP could also be considered as a limitation of our study, because it involve the researcher’s decision on some steps, as the choice of food groups, number of DP, etc. In addition, associations between the DP and %BF both measured at age 18 years should be interpreted with caution, considering the possibility of reverse causality. This is a bias that occurs when exposure and outcome are measured in the same time point. We cannot ruled out that adolescents who have higher %BF may be are those trying to follow a diet to control body weight and report a restrictive eating habits In spite of these limitations, we highlight some strengths of this study regarding its design, as the associations between DP and body fat were tested using data from a large and long running birth cohort study. In addition, the %BF was measured using a modern and precise method [31].

## Conclusions

A greater average %BF was found among those who presented “Dieting” DP tracking over time or changed to a “Dieting” DP or “Fish, fast food and alcohol” DP at age 18 years. Individuals who adhered to these DP constitute a potential target for strategies aiming to promote the adoption of healthy food choices and prevent excessive fat accumulation. Further studies are need to investigate the relationship between tracking and change of the DP during adolescence and fat accumulation later in life, especially in low and middle-income settings, also evaluating associations with lifestyle features associated with a positive energy balance. These results may support public health policies and strategies focused on improving dietary habits of adolescents and young adults.
